# Hosts of avian brood parasites have evolved egg signatures with elevated information content

**DOI:** 10.1098/rspb.2015.0598

**Published:** 2015-07-07

**Authors:** Eleanor M. Caves, Martin Stevens, Edwin S. Iversen, Claire N. Spottiswoode

**Affiliations:** 1 Department of Zoology, University of Cambridge, Downing Street, Cambridge CB2 3EJ, UK; 2 Centre for Ecology and Conservation, College of Life and Environmental Sciences, University of Exeter, Penryn Campus, Penryn, Cornwall TR10 9FE, UK; 3 Department of Statistical Science, Duke University, PO Box 90251, Durham, NC 27708-0251, USA; 4 DST-NRF Centre of Excellence at the Percy FitzPatrick Institute, University of Cape Town, Cape Town, Rondebosch 7701, South Africa

**Keywords:** avian vision, brood parasitism, coevolution, entropy, information theory, signals

## Abstract

Hosts of brood-parasitic birds must distinguish their own eggs from parasitic mimics, or pay the cost of mistakenly raising a foreign chick. Egg discrimination is easier when different host females of the same species each lay visually distinctive eggs (egg ‘signatures’), which helps to foil mimicry by parasites. Here, we ask whether brood parasitism is associated with lower levels of correlation between different egg traits in hosts, making individual host signatures more distinctive and informative. We used entropy as an index of the potential information content encoded by nine aspects of colour, pattern and luminance of eggs of different species in two African bird families (Cisticolidae parasitized by cuckoo finches *Anomalospiza imberbis*, and Ploceidae by diederik cuckoos *Chrysococcyx caprius*). Parasitized species showed consistently higher entropy in egg traits than did related, unparasitized species. Decomposing entropy into two variation components revealed that this was mainly driven by parasitized species having lower levels of correlation between different egg traits, rather than higher overall levels of variation in each individual egg trait. This suggests that irrespective of the constraints that might operate on individual egg traits, hosts can further improve their defensive ‘signatures' by arranging suites of egg traits into unpredictable combinations.

## 1. Introduction

Many kinds of interactions between and among species require individuals to recognize one another or to distinguish self from non-self. For example, social animals must recognize other group members [1–3], and hosts must distinguish mimetic parasites or pathogens from themselves [4,5]. Selection can thus favour individually distinctive phenotypes, sometimes resulting in ‘signature’-like variation that allows most individuals in a population to be distinguished from one another [6]. In the hosts of avian brood parasites, selection for individual distinctiveness might result from their need to distinguish their own eggs from those of mimetic parasites [7,8]. Parasitic mimicry evolves in response to hosts rejecting mismatched eggs from their nests, and hosts are more likely to detect and reject parasitic eggs the more they differ in appearance from their own [9–11]. The greater the variation in egg phenotype among individual host females, the more distinctive the clutch of any one female, and consequently the harder it is for a brood parasite accurately to mimic it [7,12]. In birds’ eggs, such individual ‘signatures' of identity can be composed of suites of traits including eggshell colour, luminance and different aspects of pattern [11,13]. Variation in such traits leads to greater discrepancy on average between host and parasitic eggs, increasing a host's likelihood of rejecting a parasitic egg. Species that interact heavily with brood parasites should therefore be expected to show greater levels of variation in egg appearance than species that do not, but comparative tests of this prediction have found mixed results (e.g. [7,14,15]).

However, diversification in egg traits between the clutches of different females could be limited by competing selection pressures, such as camouflage [16], structural strength [17], protection against solar radiation [18] and avoiding overlap with areas of phenotypic space already occupied by other hosts and their corresponding specialist parasites [19]. Moreover, mechanistic constraints will prevent ever more extreme values of each pattern and colour trait from evolving [13,16,20]. Both kinds of constraints should limit the extent to which each egg trait can diversify over evolutionary time in the face of ever-improving parasitic mimicry. Under such constraints, how can a female increase the distinctiveness of her eggs? Distinctiveness of any given female's eggs should be maximized when different traits contributing to egg appearance are uncorrelated with one another at the population level, resulting in less predictable egg phenotypes. This generates the largest number of unique individual phenotypic combinations [2,21] and provides multiple independent cues or signal components [22], which should facilitate recognition of a female's own eggs and detection of a parasite's.

**Figure 1.**
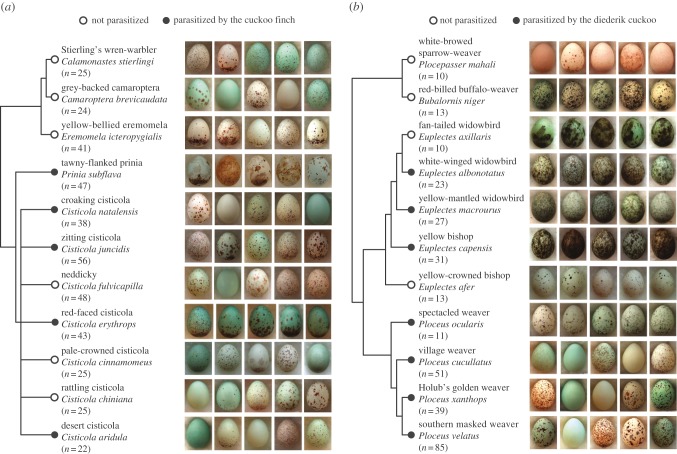
Diversity of egg phenotypes and phylogenetic relationships within each of the two host families studied here, (*a*) Cisticolidae warblers and (*b*) Ploceidae weavers. Open and closed circles, respectively, indicate unparasitized species and species parasitized by (*a*) cuckoo finch and (*b*) diederik cuckoo in our study area in Zambia. Sample sizes (clutches) and a representative selection of eggs from five clutches are shown for each species.

In support of this hypothesis, recent studies have revealed low levels of correlation among egg traits in hosts of the cuckoo finch *Anomalospiza imberbis* [11,19] and common cuckoo *Cuculus canorus* [23], potentially increasing the information about egg identity that they encode. However, neither study was able to compare these low levels of correlation to baseline levels in related, unparasitized host species. Hence, they could not specifically test whether low levels of correlation are an adaptation to parasitism. In this study, we took a comparative approach to examine sympatric parasitized and unparasitized species within two African bird families, warblers (Cisticolidae) and weavers (Ploceidae). Each family is heavily parasitized by mimetic parasites (cuckoo finches [24] and diederik cuckoos *Chrysococcyx caprius* [25], respectively), and each shows remarkable diversity in egg phenotype within and between species (figure 1). We quantified multiple egg traits (colour, luminance and several aspects of pattern) using established visual modelling approaches and metrics that have in past work predicted rejection behaviour by three warbler host species at the same study site [11,19]. Hence, this approach captures information used by hosts themselves in distinguishing their own eggs from those of parasites.

We then applied information theory [6,26] to calculate entropy as a measure of the quantity of information about egg identity potentially encoded by combinations of egg traits. Entropy, also known as uncertainty, is an information-theoretic metric that quantifies the degree of randomness in the component parts of a signal. In other words, greater entropy is associated with greater disorder or unpredictability of the signal. Shannon entropy has been used to quantify signal complexity and information content in a variety of natural systems, particularly as encoded by the syntax of acoustic signals such as bird and dolphin vocalizations [27,28]. Here, we used differential entropy (an extension of Shannon entropy for continuously measured variables) as a measure of lack of correlation (i.e. disorder) among egg traits. First, if egg ‘signatures’ composed of multiple traits evolve to maximize information content, then we predict that parasitized species should show higher entropy between different egg traits than related, unparasitized species that are not currently experiencing selection for defences against parasitism. Second, we tested the mechanism underlying this. Entropy increases as a function both of increasing absolute variation in each individual egg trait, and of decreasing correlation (i.e. greater disorder) between traits, at the population level (electronic supplementary material). We specifically wished to test whether parasitism is associated with lower correlation between traits, irrespective of absolute variation in individual traits, and therefore decomposed entropy into two variation components reflecting these two mechanisms.

## 2. Material and methods

### (a) Study system

We measured eggs from the private collection of the late J. F. R. Colebrook-Robjent (bequeathed to the Natural History Museum, Tring, UK), which were collected largely in the Choma District in southern Zambia (near 16°47′ S, 26°50′ E) over a 35-year period during the 1970s–1990s. We confined our analyses to eggs from the Choma District (within a *ca* 500 km^2^ area), with additional samples collected from the Monze and Mazabuka Districts less than 100 km away. This relatively small geographical area allows any effects of parasitism to be isolated from any potentially caused by environmental factors, while the temporal and spatial scale remain large enough to minimize the chance of pseudoreplication caused by repeated sampling of the same female.

Our dataset (summarized in figure 1) comprised 11 warbler species (six unparasitized and five parasitized by the cuckoo finch) and 11 weaver species (four unparasitized and seven parasitized by the diederik cuckoo). These are all the species occurring in our study area for which a minimum of 10 clutches was available. Ten weaver species are parasitized in our study area but three among them (*Anaplectes melanotis*, *Euplectes orix* and *Ploceus intermedius*) lay only immaculate (unpatterned) eggs, such that entropy cannot be measured on a comparable scale, and were therefore excluded. Both cuckoo finches and diederik cuckoos have evolved host-specific races or ‘gentes’, within which parasite females mimic the eggs of their specialist host species [19,25]. Some parasitic host-specific races show high levels of variability in egg appearance, corresponding to that of their particular host [19,25]. However, each parasitic race appears to lay its eggs haphazardly in nests of its specialist host, rather than targeting host females with individual egg phenotypes that match their own [11,29]. Mismatches are therefore common and parasites incur high levels of host rejection [11,29].

We classified species as parasitized if parasitism by mimetic brood parasites (cuckoo finch and diederik cuckoo for warblers and weavers, respectively) was documented from our study area in the breeding records of J. F. R. Colebrook-Robjent (*n* = 1205 breeding records for our 22 species over 38 years), which we define as parasitism status 1. However, we cannot be certain that species that are currently unparasitized here were not parasitized at some point in the past (e.g. [7,19]), although any history of parasitism in currently unparasitized species would render our results conservative (see Discussion). Three warbler species in this study (*Cisticola chiniana*, *C. cinnamomeus* and *C. fulvicapilla*) are known to be parasitized by the cuckoo finch in other parts of Africa although there are no parasitism records from Zambia, and two others have been rarely recorded as hosts of the Klaas's cuckoo *Chrysococcyx klaas* (*Camaroptera brevicaudata* elsewhere and *Eremomela icteropygialis* in our study area) [30,31]; *C. brevicaudata* may also be occasionally parasitized by other species elsewhere [30]. To guard against any uncertainty regarding the presence or absence of parasitism, we repeated all analyses with two alternative definitions of parasitism, first with these five species classified as parasitized (parasitism status 2), and second with these five species excluded (parasitism status 3). For each analysis, we pooled both families (warblers and weavers) in a single model. Using the model without phylogenetic correction, we tested for the effect of family identity and its interaction with parasitism status. We found the main effect of family identity to be highly significant, but not its interaction with parasitism status. We retained family identity as a term in all statistical models as it reduced unexplained variation, and prevented confounding the effect of parasitism status with the effect of family, as a greater proportion of weaver species than warbler species was parasitized.

### (b) Quantifying colour and pattern traits

Because avian vision differs from human vision in several important respects, we quantified egg phenotypic attributes in terms of avian visual perception (reviewed in [32]). To quantify egg colour, we measured reflectance spectra from blown eggs using an Ocean Optics USB2000 spectrophotometer, with a PX-2 pulsed xenon light source and an R400–7-UV/VIS reflectance probe (all Ocean Optics Inc., Dunedin, FL, USA). All measurements were taken with reference to a Spectralon 99% white reflectance standard (Labsphere, Congleton, UK). We used a slanted, matte black plastic sleeve to hold the egg at a constant 5 mm distance and 45° angle from the light source. We took five measurements of background colour (i.e. avoiding any overlaid markings) from each egg and used the mean in analyses. We calculated irradiance using the d65 standard measure of daylight illuminance, because irradiance spectra do not exist for the nests of all individual study species, and because previous studies have found that modelled photon catches change little when repeating models with different measures of irradiance [33]. Data for our specific study species were lacking, so we used sensitivity data from the blue tit *Cyanistes caeruleus* to calculate photon catches (i.e. measures of how stimulated a cone type is in response to a visual scene) for avian single and double cones (following [11]), resulting in photon catch values for ultraviolet, short-wave, medium-wave and long-wave (UV, SW, MW and LW) cones, as well as a measure of luminance. The blue tit is an appropriate model species because it has a UVS visual system, like the members of both of the families studied here [34], and because visual systems appear to be conserved among the Passerida, at least in terms of spectral sensitivity [34,35]. Cone catch values were standardized to remove variation in absolute brightness, such that the standardized cone catch values sum to one.

To quantify egg pattern, we analysed digital photos using a ‘granularity’ analysis [36], as previously used to examine egg pattern [11,19,23]. Photos were taken with a Fuji Finepix S7000 camera outdoors under a foil-lined umbrella (using the matte not shiny side) to reduce shadows, and including a grey standard of known reflectance (Macbeth ColorChecker, X-Rite, MI, USA). Pattern perception in birds is primarily a function of achromatic (luminance) vision [37], so following previous studies we extracted the medium-wave colour channel to generate an achromatic image for pattern analyses [23]. Achromatic images were rescaled to 50 pixels mm^−1^ and calibrated to linearize the relationship between radiance and the colour value recorded in each image before reflectance values were obtained via equalization with respect to the grey standard [38]. We then used custom MATLAB (Mathworks, Natick, MA, USA) programs and its Image Processing Toolbox for both image and pattern analysis. Within a single egg, three identically sized regions (one each from the wide, middle and narrow region of the egg) were selected, maximizing the area analysed. The exact size of the sampled regions differed between eggs owing to egg size and shape variation. We then analysed pattern markings within the selected regions using a granularity analysis using previously published protocols [23,36]. Briefly, this uses a fast Fourier transform followed by application of seven octave-wide, isotropic band-pass filters to create a new set of images, each of which captures pattern information at different spatial scales. This broadly relates to the way vertebrate spatial vision works, which operates by ‘decomposing’ the elements of a scene into different spatial frequencies [23]. Attributes captured using this method have previously been shown to predict egg rejection by three of our focal Cisticolidae species [19].

Using the resultant granularity spectrum, following previous studies [11,23], we measured three aspects of egg pattern: (i) predominant marking size, as reflected by the filter size that captured the most information; (ii) the contribution of the predominant marking size to the overall pattern, as reflected by the proportion of total energy captured by the main filter size; and (iii) the degree of contrast between egg pattern markings and background colour, as reflected by the total energy contained across all images. Finally, again following [11,23], we used thresholding to transform each image into a binary format (1 = markings; 0 = ground colour), to calculate (iv) the proportion of the egg's surface, on average across the three selected regions, that was covered with pattern markings, and (v) pattern dispersion, the difference in pattern proportion between the poles of the egg. Following [19], we standardized each pattern variable, and luminance, by expressing it as a proportion of its maximum value across all species.

### (c) Entropy as an estimate of correlation among traits

Entropy (termed ‘differential entropy’ when applied to continuously measured variables) is an information-theoretic measure of variability in a signal [21,26] and yields a univariate, composite measure of variation in a population: when entropy of a signal is zero, all members of the population have the same value. High entropy is associated with high unpredictability because of higher randomness or greater disorder among its component parts. When entropy is calculated for a multi-dimensional dataset, entropy will increase with an increase in the scale of variation in one or more dimensions; it will also increase as the strength of the correlation among one or more pairs of variables decreases towards zero [39]. Entropy cannot be calculated when any one variable can be expressed as a linear combination of the others ([40]; electronic supplementary material), such as in our data as the cone catch measures were standardized to sum to one, in order to eliminate noise introduced by luminance differences between cone catches. We therefore calculated entropy after removing one of the cone catch values. We arbitrarily removed the LW cone catch in the main text analyses, but repeated all analyses with each of the other cone catches removed in turn. This did not change any conclusions (electronic supplementary material, tables S2–S4); total entropy is the same regardless of which cone catch is removed, but the relative contributions of the variance and correlation components may differ. For example, if the omitted cone catch is swapped for another with smaller marginal variance, the relative contribution of the variance component will increase and will be offset by a decrease in the correlation component (electronic supplementary material).

We calculated a species-specific value of entropy from nine phenotypic traits (luminance, UV, MW and SW cone catches, and five measures of pattern as defined above). We considered these traits to have a species-specific multivariate normal distribution; as such, entropy depends only on the population covariance matrix, through the log of its determinant, and on the number of traits (or dimensions) [40]. Because the covariance matrix can be written as a product of the population correlation matrix and a diagonal matrix of trait standard deviations [41], entropy can be decomposed into separate, additive contributions from trait correlations and trait variances (see electronic supplementary material, §2, for details). In short, entropy decreases with the degree of correlation among traits, and increases with the degree of variation in each individual trait.

Because eggs within clutches are non-independent, we randomly selected one egg per clutch for analysis. We calculated entropy for each species (electronic supplementary material, table S1) and used linear regression to test the effects of parasitism status (parasitized versus unparasitized) and family membership (Cisticolidae versus Ploceidae). Entropy is a property of a population distribution, and we estimate species-specific entropy by replacing the population covariance matrix by the sample covariance. Therefore, the accuracy of the sample estimate of entropy depends on the sample size (here, the number of clutches measured for a given species); the variance of its sampling distribution is approximately inversely proportional to the sample size [42]. To account for this, we repeated all linear model analyses both with and without weighting the model by sample size.

### (d) Entropy decomposition

We specifically wished to test the prediction that selection from brood parasites results in egg traits being less correlated with (i.e. less predictable from) one another, irrespective of levels of overall phenotypic variation. As noted above, entropy is a function both of correlations among different traits and of the absolute degree of variation in each trait. To distinguish the two, we examine a shifted and scaled version, Ĥ, of entropy and the contributions that variance and correlation make to it (Ĥ_Var_ and Ĥ_Cor_, respectively; details in electronic supplementary material, Methods §2 and equation 3). Because entropy increases with an increase in the scale of variation in one or more dimensions (via Ĥ_Var_), it should increase with the absolute degree of variation among clutches of a given species. Species-level entropy will also increase with the second term, Ĥ_Cor_, which reflects the degree of correlation among egg traits. We therefore separately examined the variance (Ĥ_Var_) and correlation (Ĥ_Cor_) components of (scaled) entropy (Ĥ) in relation to parasitism status, in order to distinguish their relative contributions to total entropy; both are predicted to be greater in parasitized species.

### (e) Phylogenetic comparative analyses

We checked for any effect of shared phylogenetic history using phylogenetic generalized least squares (PGLS). We compiled cladograms for the Cisticolidae and the Ploceidae using published phylogenies [43–45], supplemented by S. Andersson & M. Prager (2013, unpublished data, University of Gothenburg) for the genus *Ploceus*. Phylogenetic information for *Cisticola* was incomplete so we collapsed this genus to a polytomy. Because our cladogram (figure 1) was compiled from multiple sources, branch lengths were unknown and so estimated with the R [46] package ape, using Grafen's method [47] which defines the age of each node as one less than the number of species arising from it. We used the R package caper to carry out PGLS analyses testing our main hypothesis, as well as to estimate the degree of phylogenetic dependence in entropy (using Pagel's *λ* [48], where 0 = phylogenetic independence and 1 = direct covariance with phylogenetic structure).

## 3. Results

### (a) Do parasitized species show greater entropy in egg traits than unparasitized species?

**Figure 2.**
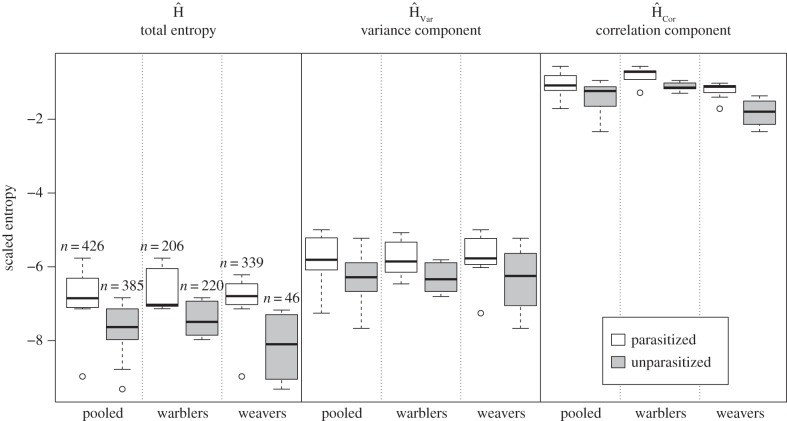
Total entropy (Ĥ), the variance contribution to entropy (Ĥ_Var_) and the correlation contribution to entropy (Ĥ_Cor_) in egg traits in relation to parasitism status for all species pooled, and for warblers and weavers separately. Whiskers indicate ranges. The numbers above the bars in the Ĥ column represent the sample size of clutches for all species combined; sample sizes in the Ĥ_Var_ and Ĥ_Cor_ columns are identical.

Table 1 presents summary statistics for entropy calculations, and table 2 presents results from weighted multiple regression and unweighted PGLS models for the total entropy measure Ĥ; results from unweighted regression models were comparable to their weighted counterparts. All analyses were adjusted for family (warblers versus weavers). Entropy was significantly higher in parasitized than in unparasitized species (coef. ± s.e. = 1.04 ± 0.31, *t*_20_ = 3.34, *p* = 0.0034) (figure 2 shows distributions for warblers and weavers separately), and results were consistent using the alternative definitions of parasitism status (table 2). There was no evidence of any phylogenetic signal in entropy: the point estimate for *λ* was 0.00 for all definitions of parasitism status, differing significantly from one but not from zero. Results using PGLS models adjusting for family and taking phylogenetic structure into account are reported in table 2; these agreed to two decimal places with estimates from the unweighted multiple regression analyses without phylogenetic correction (not shown).

**Table 1.** Summary statistics (mean ± s.d.) for entropy (information content), and the contributions of Ĥ_Var_ and Ĥ_Cor_ to total entropy, in relation to parasitism status. Parasitism status 1: species scored as ‘unparasitized’ when no parasitism records by the focal brood parasite (cuckoo finch for warblers, diederik cuckoo for weavers) exist for our study area in Zambia (d.f. = 1, 20); parasitism status 2: species scored as ‘unparasitized’ when no parasitism records exist by any brood-parasitic species anywhere in their range (d.f. = 1, 20); parasitism status 3: species differing in parasitism status 1 and 2 (*n* = 5) omitted from analyses (d.f. = 1, 15).

	total entropy (Ĥ)		variance component (Ĥ_Var_)		correlation component (Ĥ_Cor_)	
	parasitized species	unparasitized species	parasitized species	unparasitized species	parasitized species	unparasitized species
parasitism status 1	−6.83 ± 0.81	−7.73 ± 0.81	−5.77 ± 0.66	−6.32 ± 0.67	−1.06 ± 0.32	−1.40 ± 0.44
parasitism status 2	−6.98 ± 0.76	−8.11 ± 0.91	−5.91 ± 0.64	−6.39 ± 0.88	−1.07 ± 0.27	−1.72 ± 0.43
parasitism status 3	−6.83 ± 0.81	−8.11 ± 0.91	−5.77 ± 0.66	−6.39 ± 0.88	−1.06 ± 0.32	−1.72 ± 0.43

**Table 2.** Results of linear models relating entropy to parasitism status (see table 1 legend for parasitism status definitions). In the PGLS, for each model, *λ* differed significantly from one but not from zero, indicating little to no phylogenetic signal in the model residuals.

	no phylogenetic correction; weighted by sample size and adjusted for family				PGLS analysis; unweighted			
	slope ± s.e.	*t*	*r^2^*	*p*	slope ± s.e.	*t*	*r^2^*	*p*
Ĥ (total entropy)								
parasitism status 1	1.04 ± 0.31	3.34	0.30	0.0034	0.99 ± 0.34	2.92	0.27	0.0088
parasitism status 2	1.33 ± 0.51	2.60	0.19	0.018	1.10 ± 0.44	2.50	0.20	0.022
parasitism status 3	1.47 ± 0.48	3.06	0.33	0.0086	1.19 ± 0.46	2.58	0.29	0.022
Ĥ_Var_ (variance component)								
parasitism status 1	0.63 ± 0.28	2.29	0.20	0.034	0.56 ± 0.30	1.88	0.07	0.076
parasitism status 2	0.80 ± 0.44	1.83	0.13	0.083	0.57 ± 0.38	1.51	0.018	0.15
parasitism status 3	0.89 ± 0.45	2.00	0.13	0.065	0.63 ± 0.41	1.54	0.025	0.15
Ĥ_Cor_ (correlation component)								
parasitism status 1	0.40 ± 0.10	4.10	0.57	0.0006	0.44 ± 0.12	3.69	0.56	0.0016
parasitism status 2	0.53 ± 0.17	3.16	0.47	0.0052	0.53 ± 0.15	3.57	0.55	0.0021
parasitism status 3	0.58 ± 0.16	3.66	0.63	0.0026	0.56 ± 0.16	3.53	0.59	0.0034

### (b) Are entropy differences due to absolute levels of trait variation or to correlation among traits?

As noted above, entropy is an increasing function of marginal trait variances and a decreasing function of correlations among the traits. We separately analysed the variance (Ĥ_Var_) and correlation (Ĥ_Cor_) components of variation in entropy to determine whether the effect of parasitism on entropy was concentrated on one or the other. Differences by parasitism status are evident (figure 2) in both components of variation, both overall and when adjusted for family. Both measures were significantly associated with parasitism status 1 in the weighted multiple regression analysis (table 2), but the association with Ĥ_Cor_ was stronger: the Ĥ_Var_ coefficient was estimated to be 0.63 ± 0.28 (*p* = 0.034), while the Ĥ_Cor_ coefficient was estimated to be 0.40 ± 0.10 (*p* = 0.0006). Parasitism status 2 and 3 yielded similar conclusions, as did the unweighted models with and without the phylogenetic correction (table 2). However, the association between parasitism status and Ĥ_Var_ was only significant in the primary analysis. By contrast, the association between parasitism status and Ĥ_Cor_ was significant with *p* < 0.01 for all models and all definitions of status. This implies that the greater entropy observed in parasitized species arises as both a function of specific trait assemblages in individuals (Ĥ_Cor_) and as a function of the absolute degree of variation per trait (Ĥ_Var_) in each species, but primarily the former.

## 4. Discussion

Across two families of African birds, we found that the eggs of species targeted by brood parasites showed higher levels of entropy among different components of egg appearance than did the eggs of unparasitized species. This should maximize the effectiveness of such egg ‘signatures’ by increasing the unpredictability of egg appearance between different females, and hence the information content potentially available to host parents in identifying and rejecting parasitic eggs. Entropy increases with low levels of correlation among egg traits, which have previously been noted in hosts of the common cuckoo [23] and the cuckoo finch [19]. This study supports the hypothesis that such low levels of trait correlation are a defensive adaptation, by showing that they are specifically associated with the incidence of brood parasitism. Moreover, we show that this association exists independently of trait variance, which also tended to be higher in parasitized species: we separately quantified the contributions of trait variance (Ĥ_Var_) and correlation (Ĥ_Cor_) to total entropy, and found the association with the correlation component to be stronger. This suggests that the higher levels of entropy observed in parasitized species arose primarily from specific combinations of traits within individuals. In short, high entropy allows host females to improve the individual distinctiveness of their eggs, regardless of any constraints that might operate on the diversification of each individual egg trait, such as competing selection pressures or mechanistic constraints.

In the absence of parasitism, we might plausibly expect low entropy in egg phenotypes. Egg appearance is determined in the shell gland which deposits egg colours and patterns on fully formed eggs a few hours before laying [49]. Very little is known about the developmental mechanisms which may or may not limit the potential range of colours and patterns produced by the shell gland [13], but it seems plausible to expect that certain egg traits should be inherently correlated. For example, the size of markings and the proportion of the egg's surface they cover might by default be related, as larger markings occupy greater area. Similarly, egg background colour might be expected to covary with pattern attributes, as red-brown background colours and egg patterning share a common pigmentary basis (protoporphyrin) [50]. Upregulation of protoporphyrin deposition might simultaneously influence both traits and hence generate default correlations between colour, luminance and pattern. This supports the suggestion that biological mechanisms exist which keep correlations among egg traits higher than expected by chance, underlining the role of parasitism in driving up levels of entropy.

We have assigned species in this study as parasitized or unparasitized based on currently observed incidence of parasitism, but it is possible that currently unparasitized species may have been parasitized in the past. For example, the same unparasitized rattling cisticola *Cisticola chiniana* population has been experimentally shown to have strong egg rejection behaviour, suggestive of past interactions with a now locally extinct host-race of cuckoo finches [19]. This species also showed the next highest value of Ĥ_cor_, after four currently parasitized warbler species (electronic supplementary material, table S4), consistent with a history of parasitism. We tried to guard against the possibility of past parasitism by investigating the effect of those five ‘unparasitized’ species which are parasitized in other parts of Africa and/or by a different brood parasite in Zambia. This did not change the significance of any results, either when excluding those species entirely or when treating them as parasitized (table 2). Nonetheless, any such effect should render our results conservative as previous interactions with brood parasites should have elevated entropy in currently unparasitized species, contrary to our prediction and findings. Moreover, host egg traits can respond quickly to changes in selection from brood parasites [20,51]; for example, variability in village weaver *Ploceus cucullatus* egg appearance has been shown to dwindle rapidly (less than 200 years) in two populations released from brood parasitism [51]. More broadly, there is good comparative evidence that egg traits are highly evolutionarily labile in birds [49,52], supported by our finding of low phylogenetic signal in entropy.

These findings may have wider implications for the other ecological contexts in which individually unique phenotypes have evolved. In particular, our study supports previous work that has highlighted the importance of multi-component signals of individual identity in nature [2,21]. For example, there is strong experimental evidence that multi-component visual signals can generate large numbers of distinct signatures in the paper wasp *Polistes fuscatus*, which uses both facial and abdominal markings visually to identify individual nest-mates [1]. Taken together with previous experimental evidence that cuckoo finch hosts use multiple aspects of egg appearance as cues to reject foreign eggs [19], the present study suggests that the interaction between egg traits similarly represents a mechanism for maximizing the number of potential individual signatures in a population.

These results support previous work on other brood-parasitic systems in suggesting that parasitism pressure can be a powerful driver of complexity in egg phenotype [13]. These studies underscore the importance of studying not only individual phenotypic traits, but also the interaction between them, which we suggest may itself function as an adaptive defence against parasitism. More broadly, this study shows how the form of multi-component signals can be shaped by selection to increase the reliability of information transfer [53]. Similar modifications in signal form that increase the information available to receivers may occur in other areas of communication; for example, species recognition and efficient signalling of other individual attributes, such as multiple messages about individual quality [22].

## Supplementary Material

Supplementary Methods and Tables

## Data accessibility

Data are available from the Dryad Digital Repository: http://dx.doi.org/10.5061/dryad.9ph5c.
