# Supplementary material for: Hosts of avian brood parasites have evolved egg signatures with elevated information content
Source: Proc Biol Sci. 2015 Jul 7;282(1810):20150598. doi: 10.1098/rspb.2015.0598 (PMC4590476; doi:10.1098/rspb.2015.0598)
Supplement: Supplementary Methods and Tables [file rspb20150598supp1.pdf]

ELECTRONIC SUPPLEMENTARY MATERIAL

1 Supplementary Methods

(a) Definition of entropy

Entropy is an information-theoretic measure of variability that finds numerous applications in statistics [1] and, increasingly, in biology [2]. In our manuscript, we employ it as a summary of species–level phenotypic variation and as a form of evidence for adaptation in hosts of brood–parasitic birds. In particular, we expect that parasitised species will have lower correlation, on average, among their egg traits. This would correspond to their having higher entropy (greater disorder) than the traits of non–parasitised species' eggs. To this end, it is convenient to think of the set of egg traits as having a species–specific normal distribution. The differential entropy (the measure of entropy used for continuous variables) of a p–dimensional multivariate normal random variable $\mathbf{Y} = (Y_1, \ldots, Y_p)$ with mean μ and covariance matrix Σ is

$$H(Y) \;=\; 0.5 \log\Big((2\pi e)^p |\Sigma|\Big), \tag{1}$$

(Theorem 9.4.1, p. 230 in ref. [1]) where $|\Sigma| = \det(\Sigma) = \prod_i^p e_i^*$ (Result 6B, p. 243 in ref. [3]) where $e_1^*, \ldots, e_p^*$ are the eigenvalues of Σ and e_i^* is the variance explained by principal component i (Equation 8–5, p. 363 in ref. [4]).

(b) Variance–correlation decomposition

The variance–covariance matrix can be rewritten as $\Sigma = SRS$, a matrix product involving a diagonal matrix of marginal trait–level standard deviations, $S = \mathrm{diag}(\sigma_1, \ldots, \sigma_p)$, and the trait correlation matrix R (Equation 2–36, p. 59 in ref. [4]). A consequence of this is that the determinant of Σ can be factored as the product of two terms, one involving only the marginal trait variances and the other the trait correlations:

$$|\Sigma| \;=\; |SRS| \;=\; |S^2|\,|R| \tag{2}$$

(Property 9, p. 206 in ref. [3]). This allows us to decompose entropy into the sum of three terms, a constant and separate terms representing total variation and correlation, respectively. In particular,

$$\begin{aligned} H(Y) &= 0.5 \log\Big((2\pi e)^p\Big) \;+\; 0.5 \log\Big(|\Sigma|\Big) \\ &= \frac{p}{2} \log(2\pi e) \;+\; \frac{1}{2} \sum_{i=1}^{p} \log(\sigma_i^2) \;+\; \frac{1}{2} \sum_{i=1}^{p} \log(e_i), \end{aligned}$$

where $e_1, \ldots, e_p$ are the eigenvalues of R. Only the latter two terms vary from species to species when p, the number of traits, does not vary and all terms scale with the number of traits. Hence, for purposes of our analysis, we ignore the constant term and scale by the number of traits, i.e. characterize variation in

$$\hat{H}(Y) \;=\; \left(\frac{2}{p} H(Y) - \log(2\pi e)\right) \;=\; \frac{\sum_{i=1}^{p} \log(\sigma_i^2)}{p} + \frac{\sum_{i=1}^{p} \log(e_i)}{p} \;=\; \hat{H}_{\mathrm{Var}}(Y) + \hat{H}_{\mathrm{Cor}}(Y). \tag{3}$$

This standardized measure of entropy is the sum of the average logged trait variance, $\hat{H}_{\mathrm{Var}}(Y)$, and the average of the logged eigenvalues of the correlation matrix, $\hat{H}_{\mathrm{Cor}}(Y)$. A species that

adapts by exploiting a greater range of variation in a given trait while leaving trait correlations fixed will increase entropy by increasing the first term (note that $H(Y)$ and $\hat{H}(Y)$ are increasing functions of the marginal variance terms, σ_i^2), while one that adapts by reducing correlations among the traits, leaving variances fixed, will increase entropy by increasing the second term. In what follows, we show how reduced dependence among traits corresponds to increased entropy.

First, note that the differential entropy of Y can be decomposed into the sum of conditional and a marginal differential entropies, $H(Y) = H(Y_1 \,|\, Y_2, \ldots Y_p) + H(Y_2, \ldots Y_p)$, the former relating to the conditional distribution of Y_1 given all other Y's and the latter relating to the marginal (to Y_1) distribution of the remaining Y's (Equation 9.33, p. 230 in ref. [1]). We will use this decomposition to show that differential entropy increases with a weakening of the correlation structure in the form of reduced dependence of one trait (arbitrarily labeled Y_1) on the remaining traits. We assume that the distribution of the remaining traits, and hence $H(Y_2, \ldots, Y_p)$, remains unchanged.

Because Y has a multivariate normal distribution, it follows that the conditional distribution of Y_1 given $Y_{>1} = (Y_2, \ldots Y_p)$ is a multivariate linear regression of Y_1 on $Y_{>1}$. In particular, it is normal with mean $\mu_1 + \Sigma_{1,>1}\Sigma_{>1,>1}^{-1}(Y_{>1} - \mu_{>1})^T$ and variance $\sigma_1^2 - \Sigma_{1,>1}\Sigma_{>1,>1}^{-1}\Sigma_{1,>1}^T$, where $\Sigma_{1,>1} = (\sigma_{1,2}^2, \ldots, \sigma_{1,p}^2)$, the vector of covariances between trait one and traits two through p (Result 4.6, p. 135 in ref. [4]). Hence, by Equation 1,

$$H(Y_1 \,|\, Y_2, \ldots Y_p) \;=\; 0.5\log(2\pi e) \;+\; 0.5\log\Big(\sigma_1^2 \;-\; \Sigma_{1,>1}\Sigma_{>1,>1}^{-1}\Sigma_{1,>1}^T\Big).$$

Note that the covariance, $\sigma_{1,i}^2$, between traits one and i can be expressed as $\sigma_1\sigma_i\rho_{1,i}$, a product of marginal standard deviations and the correlation between traits one and i (Equation 2–33, p. 58 in ref. [4]).

Suppose that some bird species have adapted by reducing dependence of trait one on the remaining traits such that the marginal variance of Y_1, σ_1^2, as well as the distribution of $Y_{>1}$, remain the same. In particular, suppose that the correlations between trait one and traits two through p are diminished by a constant factor $0 \leq \pi < 1$, so that

$$\Sigma_{1,>1}^{\text{adapt}} \;=\; (\sigma_1\sigma_2\pi\rho_{1,2}, \ldots, \sigma_1\sigma_p\pi\rho_{1,p}) \;=\; \pi\Sigma_{1,>1}.$$

For such a species,

$$H^{\text{adapt}}(Y_1 \,|\, Y_2, \ldots Y_p) \;=\; 0.5\log(2\pi e) \;+\; 0.5\log\Big(\sigma_1^2 \;-\; \pi^2\Sigma_{1,>1}\Sigma_{>1,>1}^{-1}\Sigma_{1,>1}^T\Big).$$

Note that, since $\Sigma_{>1,>1}$ is a positive definite covariance matrix, the quadratic form

$$Q \;=\; \Sigma_{1,>1}\Sigma_{>1,>1}^{-1}\Sigma_{1,>1}^T \geq 0$$

[Result 6O, p. 288][3]. Further, since $0 \leq \pi^2 < 1$,

$$H^{\text{adapt}}(Y_1 \,|\, Y_2, \ldots Y_p) \;>\; H(Y_1 \,|\, Y_2, \ldots Y_p).$$

Hence, the conditional differential entropy for the adapting species is greater than that of its non–adapting conspecifics and therefore its differential entropy, $H^{\text{adapt}}(Y)$, is greater as a result.

(c) Linear constraints among phenotypic measures

Differential entropy is not defined when one or more constraints are present among the variables, as is the case here, where the cone catch measures are standardized to sum to one. This is

because the variables do not have a proper density and the integral is not defined (Remark, p. 225 in ref. [1]). There is no variation in one dimension of the phenotype matrix when all variables that are co–standardized are included; it is akin to including a phenotype measurement that is constant. The solution is to restrict attention to the dimensions of the phenotype matrix that do vary. This can be accomplished here by removing any one of the cone catch variables. In what follows, we show that the value of the total entropy $\hat{H}(Y)$ does not depend on which cone catch variables are removed but that the balance between the variance and correlation components is sensitive to this choice. For this reason, we repeated our analysis with each of the four cone catch variables removed, in turn.

Denote the multivariate phenotype variable by $\mathbf{Y}$, the elements in that matrix containing the cone catch measures Y_{LW}, Y_{MW}, Y_{SW}, and Y_{UV} and the remaining phenotype elements $Y_1, \ldots, Y_q$ so that $\mathbf{Y} = (Y_{\mathrm{LW}}, Y_{\mathrm{MW}}, Y_{\mathrm{SW}}, Y_{\mathrm{UV}}, Y_1, \ldots, Y_q)$. The constraint is such that $Y_{\mathrm{LW}} + Y_{\mathrm{MW}} + Y_{\mathrm{SW}} + Y_{\mathrm{UV}} = 1$. Hence variation in a given cone catch can be determined from the others by a simple linear relationship. In words, variation in any one of the standardized cone catches is reflected entirely in the remaining three.

Consider, for example, an analysis of the phenotype vector with the LW cone catch removed, $\mathbf{Y}_{(-\mathrm{LW})} = (Y_{\mathrm{MW}}, Y_{\mathrm{SW}}, Y_{\mathrm{UV}}, Y_1, \ldots, Y_q)$. Because of the linear constraint, $\mathbf{Y}_{(-\mathrm{LW})}$ can be determined via simple linear transformation from the phenotype vector with any of the other standardized cone catches removed . For example, $\mathbf{Y}_{(-\mathrm{MW})} = \mathbf{C} + \mathbf{Y}_{(-\mathrm{LW})}\mathbf{A}$ where $\mathbf{C} = (1, 0, \ldots, 0)$ and where $\mathbf{A}$ is the $(q+3) \times (q+3)$ identity matrix with the first three elements of its first column replaced by -1's:

$$\mathbf{A} = \begin{pmatrix} -1 & 0 & 0 & 0 & \cdots \\ -1 & 1 & 0 & 0 & \cdots \\ -1 & 0 & 1 & 0 & \cdots \\ 0 & 0 & 0 & 1 & \cdots \\ \vdots & \vdots & \vdots & \vdots & \ddots \end{pmatrix}.$$

This linear operation has the effect of replacing Y_{MW}, the first element of $\mathbf{Y}_{(-\mathrm{LW})}$ by $Y_{\mathrm{LW}} = 1 - Y_{\mathrm{MW}} - Y_{\mathrm{SW}} - Y_{\mathrm{UV}}$, while leaving its remaining elements unchanged.

If $\mathbf{Y}_{(-\mathrm{LW})}$ is multivariate normal with covariance matrix Σ, then $\mathbf{Y}_{(-\mathrm{MW})}$ is multivariate normal with covariance matrix $\mathbf{A}^{\mathrm{T}}\Sigma\mathbf{A}$ (Result 4.3, p. 132 in ref. [4]). As noted above, the entropy of a multivariate normal phenotype vector is determined by the determinant of its covariance matrix. Note that

$$\begin{aligned} \left|\mathbf{A}^{\mathrm{T}}\Sigma\mathbf{A}\right| &= \left|\mathbf{A}^{\mathrm{T}}\right| |\Sigma| \, |\mathbf{A}| \\ &= |\Sigma| \, |\mathbf{A}|^2 \\ &= |\Sigma| \end{aligned}$$

(Property 9, p. 206 in ref. [3]) since $|\mathbf{A}| = -1$. Hence the differential entropy estimated using $\mathbf{Y}_{(-\mathrm{LW})}$ is equal to that estimated using $\mathbf{Y}_{(-\mathrm{MW})}$ or when leaving out any one of the other standardized cone catch measures (Tables S2–S4).

While entropy does not change, the relative contributions of the marginal variances and the correlations likely will (Tables S2–S4). To see this, note that any two choices of the phenotype vector will differ by only one element. In the above example, the LW component replaces the MW component; all other components remain the same. As a result, the value of $\hat{H}_{\mathrm{Var}}(Y)$ changes by a factor of

$$\Big(\log(\sigma^2_{\mathrm{LW}}) - \log(\sigma^2_{\mathrm{MW}})\Big)/p,$$

where σ^2_{LW} and σ^2_{MW} are the marginal variances of the LW and MW cone catches, respectively. This quantity will be small when the two marginal variances are similar and zero only when they are equivalent. This change is offset by a change in $\hat{H}_{\mathrm{Cor}}(Y)$ of the same magnitude, but in the opposite direction.

References

1. Cover TA, Thomas JA. 1991 *Elements of information theory.* New York: John Wiley and Sons.
2. Gatenby RA, Frieden BR. 2007 Information theory in living systems, methods, applications, and challenges. *Bull. Math. Biol.* **69**, 635-657.
3. Strang G. 1993 *Introduction to linear algebra.* Wellesley, MA: Wellesley-Cambridge Press.
4. Johnson RA, Wichern DW. 1982 *Applied multivariate statistical analysis.* Englewood Cliffs, NJ: Prentice-Hall.

2 Supplementary Tables

Table S1: : $\hat{\mathrm{H}}$, $\hat{\mathrm{H}}_{\mathrm{Var}}$ and $\hat{\mathrm{H}}_{\mathrm{Cor}}$ for each of our study species. Parasitism status here refers to Parasitism Status 1, as outlined in the main text.

Species	Parasitism status	Total entropy ($\hat{\mathbf{H}}$)	Variance component ($\hat{\mathbf{H}}_{\mathbf{Var}}$)	Correlation component ($\hat{\mathbf{H}}_{\mathbf{Cor}}$)
**Warblers (Cisticolidae):**				
Calamonastes stierlingi	Unparasitised	-7.85	-6.55	-1.30
Camaroptera brevicaudata	Unparasitised	-7.14	-6.12	-1.02
Cisticola aridula	Parasitised	-7.14	-5.85	-1.28
Cisticola chiniana	Unparasitised	-6.84	-5.89	-0.95
Cisticola cinnamomeus	Unparasitised	-7.84	-6.67	-1.18
Cisticola erythrops	Parasitised	-7.03	-6.46	-0.57
Cisticola fulvicapilla	Unparasitised	-6.93	-5.81	-1.12
Cisticola juncidis	Parasitised	-5.76	-5.07	-0.69
Cisticola natalensis	Parasitised	-7.07	-6.15	-0.93
Eremomela icteropygialis	Unparasitised	-7.97	-6.80	-1.17
Prinia subflava	Parasitised	-6.05	-5.33	-0.72
**Weavers (Ploceidae):**				
Anaplectes melanotis	Parasitised	-11.5	-10.27	-1.19
Bubalornis niger	Unparasitised	-7.42	-6.05	-1.37
Euplectes afer	Unparasitised	-9.31	-7.67	-1.65
Euplectes albonotatus	Parasitised	-6.80	-5.77	-1.02
Euplectes axillaris	Unparasitised	-7.17	-5.23	-1.94
Euplectes capensis	Parasitised	-6.52	-5.36	-1.16
Euplectes macrourus	Parasitised	-7.14	-6.02	-1.12
Euplectes orix	Parasitised	-11.0	-9.94	-1.03
Plocepasser mahali	Unparasitised	-8.78	-6.44	-2.34
Ploceus cucullatus	Parasitised	-6.91	-5.85	-1.06
Ploceus intermedius	Parasitised	-12.8	-11.1	-1.61
Ploceus ocularis	Parasitised	-8.97	-7.26	-1.71
Ploceus velatus	Parasitised	-6.22	-5.10	-1.14
Ploceus xanthops	Parasitised	-6.40	-5.00	-1.40

Table S2: Summary statistics (mean ± s.d.) for entropy, and the contributions of H_{Var} and H_{Cor} to total entropy, in relation to parasitism status with UV, MW, and SW cone catches removed (see Table 1 legend for parasitism status definitions).

	Total entropy ($\hat{H}$)		Variance component ($\hat{H}_{Var}$)		Correlation component ($\hat{H}_{Cor}$)	
	parasitized species	unparasitized species	parasitized species	unparasitized species	parasitized species	unparasitized species
**UV cone catch removed**						
Parasitism status 1:	-6.83 ±0.81	-7.73 ±0.81	-5.60 ±0.66	-6.26 ±0.67	-1.23 ±0.37	-1.46 ±0.52
Parasitism status 2:	-6.98 ±0.76	-8.11 ±0.91	-5.80 ±0.67	-6.24 ±0.91	-1.18 ±0.33	-1.87 ±0.42
Parasitism status 3:	-6.83 ±0.81	-8.11 ±0.91	-5.60 ±0.66	-6.24 ±0.91	-1.23 ±0.37	-1.87 ±0.42
**MW cone catch removed**						
Parasitism status 1:	-6.83 ±0.81	-7.73 ±0.81	-5.57 ±0.68	-6.24 ±0.67	-1.26 ±0.29	-1.48 ±0.69
Parasitism status 2:	-6.98 ±0.76	-8.11 ±0.91	-5.81 ±0.71	-6.12 ±0.90	-1.18 ±0.28	-1.99 ±0.62
Parasitism status 3:	-6.83 ±0.81	-8.11 ±0.91	-5.57 ±0.68	-6.12 ±0.90	-1.26 ±0.29	-1.99 ±0.62
**SW cone catch removed**						
Parasitism status 1:	-6.83 ±0.81	-7.73 ±0.81	-5.51 ±0.61	-6.06 ±0.65	-1.33 ±0.36	-1.66 ±0.46
Parasitism status 2:	-6.98 ±0.76	-8.11 ±0.91	-5.66 ±0.60	-6.10 ±0.86	-1.32 ±0.31	-2.00 ±0.37
Parasitism status 3:	-6.83 ±0.81	-8.11 ±0.91	-5.51 ±0.61	-6.10 ±0.86	-1.33 ±0.36	-2.00 ±0.37

Table S3: Results of linear models, with UV cone catch removed, relating entropy to parasitism status (see Table 1 legend for parasitism status definitions). In the phylogenetic generalised least squares (PGLS), for each model lambda differed significantly from one but not from zero, indicating little to no phylogenetic signal in the model residuals.

	No phylogenetic correction; weighted by sample size and adjusted for family				PGLS analysis; unweighted			
	slope ± SE	t	r^2	p	slope ± SE	t	r^2	p
**$\hat{H}$(total entropy)**								
Parasitism status 1:	1.04 ±0.31	3.34	0.30	0.0034	0.99 ±0.34	2.92	0.27	0.0088
Parasitism status 2:	1.33 ±0.51	2.60	0.19	0.018	1.10 ±0.44	2.50	0.20	0.022
Parasitism status 3:	1.47 ±0.48	3.06	0.33	0.0086	1.19 ±0.46	2.58	0.29	0.022
**$\hat{H}_{Var}$(variance component)**								
Parasitism status 1:	0.77 ±0.28	2.75	0.30	0.013	0.65 ±0.30	2.17	0.13	0.043
Parasitism status 2:	0.77 ±0.47	1.64	0.14	0.12	0.57 ±0.39	1.46	0.02	0.16
Parasitism status 3:	0.89 ±0.46	1.94	0.12	0.072	0.65±0.41	1.57	0.03	0.14
**$\hat{H}_{Cor}$(correlation component)**								
Parasitism status 1:	0.27 ±0.12	2.18	0.46	0.042	0.35 ±0.14	2.50	0.50	0.022
Parasitism status 2:	0.56 ±0.17	3.36	0.58	0.0033	0.53 ±0.16	3.42	0.59	0.003
Parasitism status 3:	0.58 ±0.18	3.19	0.57	0.0066	0.54 ±0.18	3.08	0.54	0.008

Table S4: Results of linear models, with MW cone catch removed, relating entropy to parasitism status (see Table 1 legend for parasitism status definitions). In the phylogenetic generalised least squares (PGLS), for each model lambda differed significantly from one but not from zero, indicating little to no phylogenetic signal in the model residuals. Removal of the MW cone reduces the contribution of brownish-green eggs, such as those of *Eremomela icteropygialis*.

	No phylogenetic correction; weighted by sample size and adjusted for family				PGLS analysis; unweighted			
	slope ± SE	t	r^2	p	slope ± SE	t	r^2	p
**$\hat{H}$(total entropy)**								
Parasitism status 1:	1.04 ±0.31	3.34	0.30	0.0034	0.99 ±0.34	2.92	0.27	0.0088
Parasitism status 2:	1.33 ±0.51	2.60	0.19	0.018	1.10 ±0.44	2.50	0.20	0.022
Parasitism status 3:	1.47 ±0.48	3.06	0.33	0.0086	1.19 ±0.46	2.58	0.29	0.022
**$\hat{H}_{Var}$(variance component)**								
Parasitism status 1:	0.84 ±0.27	3.13	0.35	0.006	0.64 ±0.30	2.14	0.14	0.045
Parasitism status 2:	0.73 ±0.48	1.53	0.13	0.14	0.46 ±0.40	1.16	0.005	0.26
Parasitism status 3:	0.88 ±0.45	1.94	0.11	0.073	0.56 ±0.42	1.34	0.013	0.20
**$\hat{H}_{Cor}$(correlation component)**								
Parasitism status 1:	0.19 ±0.14	1.40	0.38	0.178	0.35 ±0.16	2.22	0.49	0.039
Parasitism status 2:	0.60 ±0.17	3.51	0.58	0.0024	0.63 ±0.16	3.87	0.64	0.001
Parasitism status 3:	0.59 ±0.19	3.15	0.53	0.007	0.63 ±0.19	3.36	0.56	0.0047

Table S5: Results of linear models, with SW cone catch removed, relating entropy to parasitism status (see Table 1 legend for parasitism status definitions). In the phylogenetic generalised least squares (PGLS), for each model lambda differed significantly from one but not from zero, indicating little to no phylogenetic signal in the model residuals.

	**No phylogenetic correction; weighted by sample size and adjusted for family**				**PGLS analysis; unweighted**			
	slope ± SE	t	r^2	p	slope ± SE	t	r^2	p
**$\hat{\mathbf{H}}$(total entropy)**								
Parasitism status 1:	1.04 ±0.31	3.34	0.30	0.0034	0.99 ±0.34	2.92	0.27	0.0088
Parasitism status 2:	1.33 ±0.51	2.60	0.19	0.018	1.10 ±0.44	2.50	0.20	0.022
Parasitism status 3:	1.47 ±0.48	3.06	0.33	0.0086	1.19 ±0.46	2.58	0.29	0.022
**$\hat{\mathbf{H}}_{\mathbf{Var}}$(variance component)**								
Parasitism status 1:	0.65 ±0.26	2.50	0.25	0.022	0.55 ±0.28	1.97	0.09	0.063
Parasitism status 2:	0.73 ±0.42	1.75	0.14	0.097	0.54 ±0.36	1.51	0.019	0.15
Parasitism status 3:	0.83 ±0.42	1.99	0.13	0.067	0.61 ±0.39	1.57	0.03	0.14
**$\hat{\mathbf{H}}_{\mathbf{Cor}}$(correlation component)**								
Parasitism status 1:	0.40 ±0.11	3.40	0.50	0.003	0.44 ±0.13	3.42	0.53	0.0028
Parasitism status 2:	0.59 ±0.18	3.39	0.50	0.0005	0.56 ±0.16	3.56	0.54	0.0021
Parasitism status 3:	0.64 ±0.16	3.89	0.64	0.0016	0.59 ±0.17	3.56	0.59	0.0031
